# Pomegranate trees quality under drought conditions using potassium silicate, nanosilver, and selenium spray with valorization of peels as fungicide extracts

**DOI:** 10.1038/s41598-022-10354-1

**Published:** 2022-04-16

**Authors:** Walid F. A. Mosa, Said I. Behiry, Hayssam M. Ali, Ahmed Abdelkhalek, Lidia Sas-Paszt, Asma A. Al-Huqail, Muhammad Moaaz Ali, Mohamed Z. M. Salem

**Affiliations:** 1 Plant Production Department (Horticulture-Pomology), Faculty of Agriculture, Saba Basha, Alexandria University, Alexandria, Egypt; 2 Agricultural Botany Department, Faculty of Agriculture, Saba Basha, Alexandria University, Alexandria, 21531 Egypt; 3 Botany and Microbiology Department, College of Science, King Saud University, P.O. Box 2455, Riyadh, 11451 Saudi Arabia; 4 Plant Protection and Biomolecular Diagnosis Department, ALCRI, City of Scientific Research and Technological Applications, New Borg El Arab City, Alexandria 21934 Egypt; 5 The National Institute of Horticultural Research, Konstytucji 3 Maja 1/3, 96-100 Skierniewice, Poland; 6 College of Horticulture, Fujian Agriculture and Forestry University, Fuzhou, 350002 China; 7 Forestry and Wood Technology Department, Faculty of Agriculture (El-Shatby), Alexandria University, Alexandria, 21545 Egypt

**Keywords:** Microbiology, Plant sciences

## Abstract

The current study was performed on 8 years old "Succary" pomegranate cultivar (*Punica granatum* L.) during the 2019 and 2020 seasons. One hundred pomegranate trees were chosen and sprayed three times at the beginning of flowering, full bloom, and 1 month later with the following treatments: water as control, 0.025, 0.05 and 0.1 mg/L Se; 5 mL/L, 7.5 and 10 mL/L Ag NPs, and 0.5, 1 and 2 mg/L K_2_Si_2_O_5_. The results showed that spraying of SE, Ag NPs, and K_2_Si_2_O_5_ ameliorated the shoot length, diameter, leaf chlorophyll content, set of fruiting percentage, and fruit yield per tree and hectare compared to control through studying seasons. Moreover, they improved the fruit weight, length, and diameter, as well as total soluble solids, total, reduced, and non-reduced sugars percent, while they lessened the juice acidity percentage compared to control. The most obvious results were noticed with Se at 0.1 mg/L, Ag NPs at 10 mL/L, and K_2_Si_2_O_5_ at 2 mg/L in both experimental seasons over the other applied treatments. By HPLC analysis, peel extracts showed the presence of several bioactive compounds of catechol, syringic acid, p-coumaric acid, benzoic acid, caffeic acid, pyrogallol, gallic acid, ferulic acid, salicylic acid, cinnamic acid, and ellagic acid. The extracts applied to Melia azedarach wood showed promising antifungal activity against *Rhizoctonia solani* and were considered wood-biofingicides.

## Introduction

Pomegranate (*Punica granatum* L.) is a deciduous tree, which has been tamed for thousands of years, has a big range from cultivars and supplies opportunities for the consumers^1,2^. Its productivity can be boosted and enhanced by using numerous substances and elements. It was found that Se was enhanced greatly the development, yield and fruit quality of some fruit crops^3–5^, such as on grape, where it raised its content and the level of its nutrition, while diminishing the concentration of heavy metals particularly in European and American species^6^. Se exerts useful effects on plant growth and may works as quasi-fundamental micronutrient through change various physiological and biochemical features, therefore the plants differ greatly in their physiological and biochemical response to Se^7^. The Foliar application of Se at 0.017 g/L 6 times, with interval 10 days increased photosynthesis, yield of in pear, grape, and peach^8^.

Using nano-fertilizers is differentiated by the possibility to use in small quantity compared with conventional fertilizers^9–12^. In the recent years, a lot of interesting was given to nano fertilizers because their application promoted the development and yield of crops and lessened the chemical fertilizers usage^13–16^. By prohibiting the leakage of nutrients, as usually occurs in the case of the usage of chemical fertilizers, nano-fertilizers can be more effective in reducing the soil pollution and other environmental hazards^17,18^. Silver nanoparticles (Ag NPs), have strong biological activity^19,20^. Ag NPs influence plants by different levels^21,22^, such as germination promotion^23^, growth activation^24^, increasing the accumulation of biomass^25^, improving shoot growth^26^, and raising the pigment content^27^.

Si implementation improved the employment of water, and dry matter^28^, increased biotic and abiotic stresses’ tolerance like drought via maintaining the balance of water, improving photosynthesis process and the composition of xylem vessels during the high rates of transpiration^29–33^. The application of Si on avocadoes reduced the respiration and the production rate of ethylene and consequently increased its growth^34^, could adhere to the reverse activity of big quantities from boron^35^. Moreover, spraying date palm with silicon raised the date development, yield and quality^36^, “Hindy bisinara” mango trees^37^, banana^38^, pomegranates^39,40^, and Flame seedless grapevines^41^, “Washington” navel orange^42^. Loquat tree (*Eriobotrya japonica*, Lind.) cv. Emanual foliar treated with K_2_Si_2_O_5_ at 1.0 and 2.0%, increased the percentages of fruit set and retention and thus the yield, fruit physical and chemical characteristics, and the concentration of 2% was better than 1%^43^. Moreover, it was noticed also that the pro harvest foliar spraying of K_2_Si_2_O_5_ minimized the percentages of fruit decomposition, loss, and total acidity.

For waste valorization, peels from pomegranates have several benefits as for the extractions of bioactive compounds. Some compounds like ellagic acid derivatives, punicalagin isomers, and delphinidin, pelargonidin 3-glucosides and 3,5-diglucosides were isolated from pomegranate juice showing good antioxidant activity^44,45^. Peel extract had markedly higher antioxidant capacity than the pulp extract^46^. Hydrolyzable tannins such as punicalin, punicalagin, pedunculagin, gallic and ellagic acid were identified in peel extracts^47^. Other phytochemicals identified from the pomegranate are organic and phenolic acids (gallic, ellagic and chlorogenic^48,49^. So, this experiment was conducted to inspect the effectiveness of selenium, Ag NPs and silicon in alleviating the effect of drought and salinity and consequently increasing the vegetative development, yield and quality of pomegranate and their effects on phytochemical compositions by HPLC and their application as a wood-biofungicide.

## Materials and methods

### Experimental design

This study is complied with relevant institutional, national, and international guidelines and legislation. This study does not contain any studies with human participants or animals performed by any of the authors, where Pomegranate cv. Succary trees at the age of 8 years planted at 4 × 5 m at a private orchard at Rashid, Alexandria Governorate, Egypt through 2019 and 2020 in a sandy soil were irrigated with dripping system. The collection of Pomegranate specimens has been done under the permission from the private land owner, at Rashid, Alexandria Governorate, Egypt. Physiochemical analysis for the soil of the experiment was listed in Table 1^50^.

**Table 1:** Physiochemical analysis of the experimental soil.

Parameters	0–30	30–60	Unit
**Mechanical analysis**			
Sand	94	94	%
Silt	0	0	%
Clay	6	6	%
Textural class	Sandy	Sandy	
pH (1:2)	7.2	7.3	–
EC (1:1, water extract)	1.95	2.04	dS/m
O.M	0.7	0.7	%
CaCO_3_	0.28	0.28	%
**Soluble cations**			
Ca^2+^	8	9	meq/L
Mg^2+^	3.4	8.2	meq/L
Na^+^	3.4	3.7	meq/L
K^+^	1.3	1.4	meq/L
**Soluble anions**			
HCO_3_^−^	0.5	0.5	meq/L
Cl^-^	7.2	6.4	meq/L
SO_4_^2−^	10.44	14.05	meq/L
**Micronutrients**			
Nitrogen (N)	49	55.05	mg/kg
Phosphorus (P)	97	780	mg/kg
Potassium (K)	197	246	mg/kg

One hundred pomegranate trees were chosen and were similar in their growth and size as possible to apply the following treatments: Control (water only), Selenium (Se) (Alfa aesar®, Alfa Aesar, headquartered in Ward Hill, Massachusetts, United States) at 0.025, 0.05 and 0.1 mg/L; silver nanoparticles (Ag NPs) at 5, 7.5 and 10 mL/L; Potassium silicate (K_2_Si_2_O_5_) (Alfa aesar®) at 0.5, 1, and 2 mg/L. The chosen trees were foliar treated at the first of flowering, full bloom and 1 month later and were received the same horticultural treatments applied at the orchard. The aforementioned treatments were ordered in Randomized Complete Block Design, where each one of them contained ten replicates/trees. The influence of the aforementioned treatments was evaluated by measuring their impacts on the following:

### Vegetative parameters

Four main vegetative shoots around the trees from each side were labelled at the start of the vegetative season, and their tallness and diameter were assessed first in April and second at October. Leaf chlorophyll content in fresh leaves was assessed by chlorophyll meter (SPAD-502; Konica Minolta, Osaka, Japan) and the results were expressed in SPAD units. Fruit set percentage was assessed according to this equation:

$$Fruit\, set\left( \% \right) = \frac{{{\text{No}}. \,{\text{of}}\, {\text{fruitlets}}}}{{{\text{No}}.\, {\text{of}}\, {\text{opened}}\, {\text{flowers}}}} \times 100$$

Fruit drop percentage was estimated by accounting dropping fruits number from the middle of June till fruit harvesting time conditions of the experiment, then expressed as a percentage according to this formula:

$$Fruit \;drop \left( \% \right) = \frac{{{\text{No}}.{ }\;{\text{of}}\;{\text{ dropped}}\;{\text{ fruits}}}}{{{\text{No}}.{ }\;{\text{of }}\;{\text{set }}\;{\text{fruits }}}} \times 100$$

Fruit cracking percent was measured according to the following equation:

$$Fruit \;cracking \left( \% \right) = \frac{{{\text{Number}}\;{\text{ of}}\;{\text{ cracking}}\;{\text{ fruits }}}}{{{\text{total }}\;{\text{number }}\;{\text{of }}\;{\text{fruits }}\;{\text{on}}\;{\text{ tree}}}} \times 100$$

Fruit sun burn proportion by accounting the sunburned fruit with the respect of total number of fruits on each tree before the picking time^51^

$$Sunburn \left( \% \right) = \frac{{{\text{No}}.{ }\;{\text{ of}}\;{\text{ fruit}}\;{\text{ sunburnt }}}}{{{\text{total }}\;{\text{No}}.{ }\;{\text{of}}\;{\text{ fruits on tree}}}} \times 100$$

At the time of picking up (October), the number and weight of fruits per tree were counted.

### physical and chemical fruit characteristics

At the time of picking up, the sample of six fruits from every tree was picked up by random way to assess the fruit weight (g), tallness, diameter, volume. Fruit firmness was determined by using Magness and Taylor pressure tester (mod. FT 327 (3–27 Ibs. Made in Italy). Total soluble solids (TSS) percent was measured by using a hand refractometer (ATAGO Co. LTD., Tokyo, Japan). Total and reducing sugars were determined by Nelson arsenate—molybdate colorimetric method^52^. Non-reducing sugars were by the difference between total sugars and reducing sugars. Fruit Titratable acidity percent was determined by AOAC method^53^, where it was expressed as citric acid in g/100 ml fruit juice. TSS/acid ratio was counted. Vitamin C mg/100 mg juice was by titration with 2,6 dichloro phenol-indo-phenol^54^ and calculated as mg/100 mL of juice. Anthocyanin content was determined^55^.

### Preparation of pomegranate extracts

Peels from treated fruit with Se, Ag NPs, and K_2_Si_2_O_5_ in April 2020 were air-dried at room temperature for 2 weeks. The dried peels from pomegranate fruits were ground to a fine powder using a small laboratory mill. About 50 g from each of the dried powder peels were extracted by the soaking method^56^ with 100 mL of acetone solvent (99%) with stirring for 6 h at room temperature. After the extraction process was finished, the extract filtered through a cotton plug and then with Whatman No. 1 filter paper. The filtered extract was concentrated by evaporating the acetone solvent to have the dried peel extracts. To prepare the concentrations, the extracts were dissolved in dimethyl sulfoxide (10% DMSO), and the concentrations levels of 2%, 4%, and 6% were obtained^57^.

### HPLC analysis of phenolic compounds

The phenolic compounds from the acetone extracts of each of the pomegranate peels were identified by HPLC (Agilent 1100) was composed of binary LC pump, a UV/Vis detector, and C18 column (125 mm × 4.60 mm, 5 µm particle size). Chromatograms were obtained and analyzed using the Agilent ChemStation. The separation and identification conditions ca be found in the previous work^58^.

### Antifungal property of wood treated with extracts

Wood samples of *Melia azedarach* tree were air-dried and be ready at about 0.5 × 1 × 1 cm. Designed wood samples were autoclaved for 20 min at 121 °C, and then left to cool.*Rhizoctonia solani* (acc# MH352450) was used for the antifungal bioassay. Three wood samples were utilized for each concentration, as well as for hesta (2.5 g/L), the positive (thiophanate-methyl 70 wp) and negative (10% DMSO) controls. The antifungal action of the extracts wood-treated in terms of the inhibition percentage of fungal linear growth (IPFLG) was measured following our last works^59–62^ by utilizing the next formula; IPFLG (%) = [(G_C_ − G_T_)/G_C_] × 100, where G_C_ and G_T_ ready the average diameters of the fungal colony of control and treatment, respectively.

### Statistical analysis

Obtained data were analyzed by one-way analysis of variance^63^. A least significant difference at 0.05% was used to compare between the means of the treatments and measured with CoHort Software (Pacific Grove, CA, USA).

## Results and discussions

### Vegetative and fruit parameters

Table 2 showed that length and thickness of shoots, leaf chlorophyll, were greatly enhanced by spraying Se, Ag NP_S_ and K_2_Si_2_O_5_ with respect to control. Besides, the same measured vegetative growth parameters were greatly improved particularly with Se at 0.1 mg/L, Ag NPs at 10 mL/L and K_2_Si_2_O_5_ at 2 mg/L compared with the other applied concentrations, and K_2_Si_2_O_5_ was the superior treatment in both studying seasons.

**Table 2:** Influence of the foliar spraying of Se, Ag NP_S_ and K_2_Si_2_O_5_ on shoot length, shoot diameter, and leaf total chlorophyll in “Succary” pomegranate cultivar 2019 and 2020 seasons.

Treatments	Shoot length (cm)		Shoot diameter (mm)		Chlorophyll content SPAD (μ olm^−2^)	
	2019	2020	2019	2020	2019	2020
Control	53.75e	63.5f	0.56e	0.59e	53.37d	56.75f
0.025 mg/L Se	63.25d	73.00e	0.59de	0.64e	53.8d	62.25e
0.05 mg/L Se	70.25c	78.00 cd	0.70bc	0.76cd	63.87b	70.75d
0.1 mg/L Se	73.50bc	85.50b	0.73b	0.79bc	73.57a	78.75ab
5 mL/L Ag NPs	64.50d	74.5de	0.65cd	0.72d	57.27cd	63.4e
7.5 mL/L Ag NPs	72.75bc	82.00bc	0.70bc	0.77cd	64.35b	73.25cd
10 mL/L Ag NPs	76.75b	85.75b	0.73b	0.83b	73.70a	80.17a
0.5 mg/L K_2_Si_2_O_5_	64.00d	75.25de	0.68bc	0.74cd	58.20c	63.75e
1 mg/L K_2_Si_2_O_5_	73.00bc	84.75b	0.71bc	0.79bc	67.45b	75.65bc
2 mg/L K_2_Si_2_O_5_	85.25a	93.00a	0.79a	0.89a	76.4a	80.25a
LSD_0.05_	4.68	4.21	0.06	0.05	3.78	3.97

Means not sharing the same letter(s) within each column are significantly different according to LSD at 0.05 level of significance.

These results are in parallel with the reported results of a lot of authors, who mentioned that spraying Se is capable of raising the resistance of plants to biotic and abiotic exertion^64,65^, and lessen the oxidation stress in chloroplast^66^, thus it can enhance the development of plants^67^. Selenium plays a crucial role in physiological processes and in improving the growth parameters of orange^68^. reported that the foliar spraying of “Washington” navel orange by selenium at 0, 20, 40, 80 and 160 ppm improved significantly the shoot number, length, secondary shoots, leaf number per shoot and leaf surface area compared with control^69^. Small dose of Se can induces the growth of plant, increases the process of photosynthesis and helps in the balance of the necessary nutrient elements^70^. Foliar application of orange trees by Se at 0, 20, 40, 80 and 160 ppm raised the growth, yield, fruit quality attributes, leaf mineral content and enzymes activity, and the concentration of 40 ppm was the best results compared with the rest applied treatments^69^. The application of selenium at five different concentrations at 0, 10, 20, 40, and 80 mg L^−1^ on four different olive cultivars: “San Felice”, “Canino”, “Frantoio”, and “Moraiolo”. It was noticed that Se concentration between 10 and 40 mg L^−1^ increased fresh and dry weight of the explants and shoot lengths^71^.

With the same trend, it was reported by many authors that Ag NPs had a good influence on growth, root- shoot ratio, and root prolongation^72–75^. Spraying of saffron corms with Ag NPs at 20–60 mg/L increased significantly the number of roots, dry weight of leaves^76^, and also the growth of plants, the surface area, length of roots and shoots as well as chlorophyll content^77^. Photosynthesis, chlorophyll content, fresh weight, root and shoot length, as well as developing of seedlings in *Brassica juncea* seedlings were enhanced by Ag NPs compared with control^78^. Spraying cucumber with Ag NPs raised the number of leaves per plant and plant height^79^. Ag NPs raised leaf area, shoots and growth of plants^24,26,80,81^, content of pigments^27^, and the accumulation of biomass^25^ and improved shoot induction and proliferation. Ag NPs-foliar spraying increased plant growth attributes in terms of length of shoot, and root, and leaf area as well as leaf total chlorophyll^16^. The foliar spraying of onion with Ag NPs at 20 ppm increased plant height, total leaf and leaf content from total chlorophyll^15^. Additionally, the foliar application of Ag NPs at 10, 12.5, and 15 mL/L. positively increased the diameter of shoots, leaf surface area, leaf total chlorophyll in leaves in ‘Florida prince’ peach cultivar^82^.

Silicon plays a great role in increasing photosynthetic rate, cell division, number of pigments, the absorption and transferring of water, and root growth as well as the tolerance against biotic and abiotic exertions^29,33,83–86^. Spraying 250 ppm K_2_Si_2_O_5_on ‘Flame seedless’ grapevines increased the leaf area and the length of main shoot as well as leaf total chlorophyll as compared to control in the two seasons^41^. Moreover, the exogenous application of K_2_Si_2_O_5_ at 0.05–0.1% on ‘Grandnaine’ banana increased greatly pseudostem height, and girth, leaf surface area, green leaves number^38^. Spraying K_2_Si_2_O_5_ at 0.05, 0.1 and 0.2% on ‘Keitte’ mango trees at the start of growth, just after fruit setting and 1 month later increased shoot length, leaf number per shoot, leaf area and total chlorophylls^87^. The spraying of orange cv. Olinda Valencia with K_2_Si_2_O_5_ increased shoot length, and diameter, leaf number per shoot, leaf area surface, as well as the height, volume and diameter of tree with respect with untreated plants^88^. Potassium silicate plays an important role in minimizing the production of ethylene and chlorophyll degeneration^43^. Spraying orange trees with K_2_Si_2_O_5_ at 0.1, 0.2 and 0.3%^89^, induced an obvious improving of shoot length and thickness, leaf area, leaf nutrient, pigment contents and total chlorophyll and the utilization of 0.2% was the superior treatment compared with control and the rest applied treatments.

Table 3 clearly showed that fruit set percentage as well as fruit number were statistically enhanced by the spray of Se, Ag NPs and K_2_Si_2_O_5_, while they lessened obviously the fruit drop proportion with the respect of untreated trees. Besides, spraying 0.1 mg/L Se, 10 mL/L Ag NPs and 2 mg/L K_2_Si_2_O_5,_ extremely increased fruit set percentage and fruit number, and minimized the fruit drop percentage compared with the other applied treatments or control in the two seasons. Spraying of Se, Ag NPs and K_2_Si_2_O_5_ reduced statistically the fruit cracking and sunburn proportion, while they increased significantly the fruit yield (Table 4). Additionally, the most positive impact on the forementioned parameters were increased by the foliar application of Se at 0.1 mg/L, Ag NPs at 10 mL/L and K_2_Si_2_O_5_ at 2 mg/L with the respect of the other treatments. The most clear results were accompanied with the foliar application of K_2_Si_2_O_5_ at 2 mg/L compared with the rest applied treatments and control in both experimental seasons.

**Table 3:** Influence of the foliar spraying of Se, Ag NP_S_ and K_2_Si_2_O_5_ on the percentages of fruit set, and fruit drop as well as fruit number of “Succary” pomegranate cultivar 2019 and 2020 seasons.

Treatments	Fruit set %		Fruit drop %		Fruit number	
	2019	2020	2019	2020	2019	2020
Control	18.14h	23.95h	25.4a	22.86a	31.75e	38.00e
0.025 mg/L Se	20.92g	26.38g	21.26b	19.74b	33.75de	40.25e
0.05 mg/L Se	25.24e	34.36e	15.55de	14.49cd	37.75c	44.50cd
0.1 mg/L Se	31.30c	39.35c	13.39fg	11.55fg	41.25ab	47.75b
5 mL/L Ag NPs	23.45f	27.45g	17.74c	15.90c	36.50cd	44.00d
7.5 mL/L Ag NPs	26.33d	34.42e	15.04ef	13.64de	38.00c	45.75bcd
10 mL/L Ag NPs	32.34b	41.74b	11.82gh	10.87fg	42.00a	51.50a
0.5 mg/L K_2_Si_2_O_5_	24.55e	29.95f	17.09cd	15.56c	37.50c	45.25bcd
1 mg/L K_2_Si_2_O_5_	27.18d	37.71d	13.80efg	12.59ef	39.bc	47.25bc
2 mg/L K_2_Si_2_O_5_	40.67a	47.1a	10.95h	10.72g	44.00a	53.00a
LSD_0.05_	0.98	1.37	1.92	1.65	2.84	2.69

Means not sharing the same letter(s) within each column are significantly different according to LSD at 0.05 level of significance.

**Table 4:** Influence of the foliar spraying of Se, Ag NP_S_ and K_2_Si_2_O_5_ on fruit cracking, and sunburn percentages and fruit yield of “Succary” pomegranate cultivar during 2019 and 2020 seasons.

Treatments	Fruit cracking %		Fruit sun burn %		Yield (kg/tree)		Yield (ton/hectare)	
	2019	2020	2019	2020	2019	2020	2019	2020
Control	22.02^a^	19.43^a^	25.07a	22.12a	11.42g	14.18h	2.28g	2.84h
0.025 mg/L Se	16.47^b^	14.92^b^	22.70b	19.79b	12.73f	15.65g	2.55f	3.13g
0.05 mg/L Se	13.68^cde^	12.10^de^	20.25cd	17.13c	15.03cd	18.27e	3.01cd	3.65e
0.1 mg/L Se	11.77^ef^	10.89^ef^	19.40cd	16.67c	17.88b	21.28c	3.57b	4.26c
5 mL/L Ag NPs	15.15^bc^	13.44^c^	22.64b	19.87b	13.71ef	17.04f	2.74ef	3.41f
7.5 mL/L Ag NPs	13.50^cde^	11.96^de^	19.70cd	16.89c	15.42cd	19.08de	3.08cd	3.81de
10 mL/L Ag NPs	11.35f	10.48f	18.70de	15.99cd	18.38b	23.23b	3.68b	4.64b
0.5 mg/L K_2_Si_2_O_5_	14.19^cd^	12.54^cd^	21.12bc	18.33bc	14.51de	18.33e	2.90de	3.67e
1 mg/L K_2_Si_2_O_5_	12.42^def^	11.00^ef^	19.60cd	16.75c	15.92c	19.90d	3.18c	3.98d
2 mg/L K_2_Si_2_O_5_	11.07f	9.70f	17.55e	14.00d	21.56a	26.70a	4.31a	5.34a
LSD_0.05_	1.95	1.21	1.72	2.16	1.17	1.18	0.23	0.23

Means not sharing the same letter(s) within each column are significantly different according to LSD at 0.05 level of significance.

These results are in the same trend with the prior results^4,5^, where the application of selenium increased efficiently the yield in horticultural crops. Besides, it was found that Se raised the yield in “Valencia” orange^68^, ‘Zaghlol’ date palm^90^ and in ‘Starking Delicious’ apple cultivar^91^. Additionally, Se acts as an anti-senescent and has the ability to improve growth and developing of plants^92,93^. Furthermore, it was found that the foliar spraying of 0, 20, 40, 80 and 160 ppm Se on ‘Washington Navel’ orange trees improved significantly the obtained yield, and the best results were noticed with the usage of 40 ppm^69^ .

Spraying Ag ions reduced the abscission of flowers and the flowering buds in ‘Alstroemeria’^94^, and in orchid^95^. Ag NP from 20 to 60 ppm raised the yield in Borage^96^. The foliar applications of Ag NPs on cucumber increased number and weight of fruit per plant^79^. Ag NP exogenous spraying at 60 ppm increased the leaf area, length of shoots and roots of *Phaseolus vulgaris* and *Zea mays*^77^. It was shown that NPs significantly raised the uptaking and transferring of NPK, thus consequently they could improve the plant growth, chlorophyll content, and grain yield^80^. Spray of Onion with 20 mg/L Ag NPs raised the yield^15^, and with 10, 12.5 and 15 mL/L, pollen viability and flowers percentages of peach cv. Florida prince were improved, and thus the fruit yield^82^.

Silicon plays an important role in rising photosynthesis rate, cell division, pigments in the plants, absorption of water, tolerance of biotic and abiotic stresses and thus the final yield^33,85^. Exogenous spraying of K_2_Si_2_O_5_ at 250 ppm raised number of clusters per vine and therefore increased the final obtained yield compared with control in the two experimental seasons^41^. The exogenous spraying of ‘Grandnaine’ banana with 0.05 and 0.1% K_2_Si_2_O_5_ raised obviously the weight of bunch and hands, number of hands per bunch, number of fingers, thus consequently it improved the final yield compared with control^38^. Spraying K_2_Si_2_O_5_ at 0.05, 0.1 and 0.2% on ‘Keitte’ mango trees at the start of growth, just after fruit setting and 1 month later increased the percentages of initial fruit setting, fruit retention, fruit weight and subsequently the yield^87^. Spraying orange cv. Olinda Valencia with K_2_Si_2_O_5_ boosted the fruit yield and 2% was the eminent treatment in the two seasons^88^. Spraying loquat trees with K_2_Si_2_O_5_at 1 or 2% improved the percentages of fruit retention, fruit weight, number of fruits per cluster and thus the final yield^43^. K_2_Si_2_O_5_ sprayed at 5000 ppm enhanced the fruit weight, depleted the fruit cracking with improving the yield in ‘Wonderful’^97^ and ‘Manfalouty’ pomegranates^98^ with the respect of untreated trees. Spraying of “Washington Navel” orange trees (by 0.1, 0.2 and 0.3% K_2_Si_2_O_5_ enhanced clearly number of fruits per tree, fruit weight, thus consequently the fruit yield^89^. The premier one was K_2_Si_2_O_5_ at 0.2% that gave an increment in the average yield than the control.

Weight, volume, length, and diameter of fruit, were clearly enhanced by spraying of Se at 0.025, 0.05 and 0.1 mg/L, Ag NPs at 5, 7.5 or 10 mL/L and K_2_Si_2_O_5_ at 0.5, 1 and 2 mg/L in comparison to control (Table 5). The influence of Se, Ag NPs and K_2_Si_2_O_5_ was boosted in parallel with the raising of the applied concentrations, where the significant results were obtained with 0.1 mg/L Se, 10 mL/L Ag NPs and 2 mg/L K_2_Si_2_O_5_. The upper treatment was found with the application of K_2_Si_2_O_5_ compared with the rest applied treatment.

**Table 5:** Influence of the foliar spraying of Se, Ag NPS and K_2_Si_2_O_5_ on weight, volume, length, and diameter of “Succary” pomegranate fruit during 2019 and 2020 seasons.

Treatments	Fruit weight (g)		Fruit volume (cm^3^)		Fruit length (cm)		Fruit diameter (cm)	
	2019	2020	2019	2020	2019	2020	2019	2020
Control	359.75g	373.25g	395.00f	410.50g	6.15h	6.25h	6.78h	6.93g
0.025 mg/L Se	377.25f	388.75f	413.50e	427.25f	7.27g	7.42g	8.21g	8.40f
0.05 mg/L Se	398.25d	410.5de	435.00c	448.75^de^	7.44f	7.88e	8.43e	8.63e
0.1 mg/L Se	433.25b	445.75b	468.25b	483.75b	8.18c	8.52c	9.02c	9.38c
5 mL/L Ag NPs	375.75f	387.25f	411.25e	423.75f	7.30g	7.60f	8.29f	8.41f
7.5 mL/L Ag NPs	405.75^cd^	417.00^cd^	441.25c	455.00^cd^	7.57e	7.88e	8.44e	8.64 e
10 mL/L Ag NPs	437.75b	451.00b	475.00b	489.25b	9.31b	9.72b	10.13b	10.44b
0.5 mg/L K_2_Si_2_O_5_	386.75e	405.00e	425.00 d	442.75e	7.47f	7.69f	8.28f	8.41f
1 mg/L K_2_Si_2_O_5_	408.25c	421.25c	443.75c	459.25c	7.99d	8.15d	8.90d	9.17d
2 mg/L K_2_Si_2_O_5_	490.25a	504.00a	527.00a	542.75a	9.49a	9.89a	10.24a	10.57a
LSD_0.05_	8.14	6.56	8.99	6.89	0.07	0.13	0.06	0.08

Means not sharing the same letter(s) within each column are significantly different according to LSD at 0.05 level of significance.

Fruit firmness, grain weight and juice volume were notably improved by the foliar spraying of Se at 0.025, 0.05 and 0.1 mg/L, Ag NPs at 5, 7.5 or 10 mL/L and K_2_Si_2_O_5_ at 0.5, 1 and 2 mg/L (Table 6). The aforementioned fruit characteristics were improved parallel to the raising of the applied concentrations from Se, Ag NPs and K_2_Si_2_O_5._ The significant increments were obtained with the spraying of K_2_Si_2_O_5_ as compared to the rest applied treatments in both experimental seasons.

**Table 6:** Influence of the foliar spraying of Se, Ag NPS and K_2_Si_2_O_5_ on fruit firmness, grain weight and juice volume in “Succary” pomegranate fruit during 2019 and 2020 seasons.

Treatments	Fruit firmness (Ib/inch^2^)		Grain weight (g)		Juice volume (mL)	
	2019	2020	2019	2020	2019	2020
Control	20.50e	22.00f	207.50f	211.00h	40.50f	44.00e
0.025 mg/L Se	22.00de	23.50f	217.5e	225.25g	42.75f	45.00e
0.05 mg/L Se	23.75bcd	27.25cde	225.50d	231.75e	48.75de	50.50bcd
0.1 mg/L Se	25.75abc	30.50c	239.00c	250.00c	52.00bc	53.00bc
5 mL/L Ag NPs	22.75de	24.25ef	218.25e	226.5fg	47.00e	47.5de
7.5 mL/L Ag NPs	24.00bcd	27.75cd	232.75c	240.50d	50.00cd	51.25bcd
10 mL/L Ag NPs	25.87ab	34.00b	246.25b	254.75b	53.00b	54.50b
0.5 mg/L K_2_Si_2_O_5_	23.00cde	24.5def	219.00e	230.25ef	48.75de	50.25cd
1 mg/L K_2_Si_2_O_5_	24.25bcd	28.75c	233.00c	240.75d	52.25bc	52.00bc
2 mg/L K_2_Si_2_O_5_	28.12a	38.00a	272.75a	279.00a	57.75a	59.25a
LSD_0.05_	2.56	3.24	6.04	4.65	2.36	3.73

Means not sharing the same letter(s) within each column are significantly different according to LSD at 0.05 level of significance.

These results are in agreement with the former results on peach^99^, on pear^100^, and on pear-jujube^101^, where Se has a crucial role in increasing the maintenance of flesh firmness and delaying fruit ripening. Selenium works as an anti-senescent and helps maintain the structure and function of the cell in plants^92,93^. Besides, treating “Zaghlol” date palm with Se increased the fruit weight^90^. Spraying *Citrus Sinensis* trees with Se at 0, 20, 40, 80 and 160 ppm improved markedly pulp weight and thickness, as well as fruit diameter, height and volume and 40 ppm was the predominant one^69^. Spraying apple trees with Se at 0.5, 1, and 1.5 mg/L twice during the enlargement fruit period increased the flesh fruit firmness^91^.

Ag NPs enlarge weight, diameter and length of fruit of cucumber^79^, 20 ppm Ag NPs on onion increased its bulb weight^15^, and peach trees with 10, 12.5, and 15 mL/L. Ag NP on peach improved greatly fruit weight, volume, length, and diameter, flesh weight as well as fruit firmness^82^.

These results are in the same trend with previous results^102^, where treating grapevines cv. Flame seedless with K_2_Si_2_O_5_ at 250 ppm increased weight of berry and cluster over control. Spraying potassium silicate increased the fruit firmness in “Amal” apricot cultivar^103^, “Anna” apple^104^ and orange^105^ as compared to untreated trees. Additionally, the exogenous sprinkle of K_2_Si_2_O_5_ on banana cv. Grandnaine at 0.05–0.1% increased weight, length, and diameter of finger^38^. The foliar application of K_2_Si_2_O_5_ at 0.05, 0.1 and 0.2% on ‘Keitte’ mango trees at the start of growth, just after fruit setting and 1 month later increased fruit length, width, firmness, and the percentage of seeds and peel^87^. The average of fruit weight, volume, dimensions, shape index and juice weight as well as fruit firmness of sprayed orange trees cvs. Washington with potassium silicate were increased^106^ and Olinda Valencia^88^. With the same trend, the foliar spray of loquat trees with K_2_Si_2_O_5_ at 1 or 2% raised the fruit weight, size, length, diameter and firmness, pulp percentage, thickness, and weight^43^. Moreover, K_2_Si_2_O_5_ sprayed at 5000 ppm increased the fruit weight in pomegranate cvs. Wonderful^97^ and Manfalouty^98^ compared with untreated trees. The exogenous sprinkle of K_2_Si_2_O_5_ at 0.1, 0.2 and 0.3% boosted the fruit weight, diameters, height, volume and Fruit peel thickness in orange cv. Washington Navel^89^.

The percent of fruit total soluble solids, TSS/acidity ratio, and fruit content from vitamin C were significantly boosted by the foliar application of Se at and, Ag NPs and K_2_Si_2_O_5_, while they reduced that fruit acidity percentage compared to control (Table 7). The best results were statistically resulted from the spraying of K_2_Si_2_O_5_ followed by Ag NPs at Ag NPs at 10 mL/L.

**Table 7:** Influence of the foliar spraying of Se, Ag NPS and K_2_Si_2_O_5_ on fruit content from TSS, acidity, TSS/acidity ratio, and V.C. of pomegranate cv. Succary during 2019 and 2020 season.

Treatments	TSS %		Acidity %		TSS/Acidity		V.C. (mg/100 mL)	
	2019	2020	2019	2020	2019	2020	2019	2020
Control	12.09f	12.67f	1.11a	1.03a	10.89g	12.28g	11.21f	11.46f
0.025 mg/L Se	12.91e	13.52e	0.99b	0.87b	13.03f	15.44f	13.57e	13.33e
0.05 mg/L Se	14.15d	15.10d	0.96cd	0.8cd	14.74e	18.87de	15.71c	15.00c
0.1 mg/L Se	15.67c	16.22bc	0.89e	0.77e	17.56c	21.12c	16.78a	15.86a
5 mL/L Ag NPs	13.17e	13.02ef	0.97bc	0.81c	13.45f	16.15f	15.07d	13.33e
7.5 mL/L Ag NPs	14.22d	15.21d	0.95d	0.78de	14.92e	19.41d	15.93bc	15.21bc
10 mL/L Ag NPs	16.48b	16.73ab	0.84e	0.73f	19.59b	22.89b	16.86a	15.86a
0.5 mg/L K_2_Si_2_O_5_	13.96d	14.67d	0.96cd	0.81c	14.48e	18.09e	15.56c	13.63d
1 mg/L K_2_Si_2_O_5_	15.31c	15.90c	0.91e	0.77e	16.85d	20.59c	16.14b	15.36b
2 mg/L K_2_Si_2_O_5_	17.29a	17.20a	0.83f	0.70g	20.78a	24.54a	16.86a	15.89a
LSD_0.05_	0.44	0.56	0.02	0.02	0.58	0.81	0.41	0.24

Means not sharing the same letter(s) within each column are significantly different at 0.05 level of significance.

The proportions of total, reduced and non-reduced sugars, fruit content from anthocyanin were markedly raised by the external application of Se at 0.025, 0.05 and 0.1 mg/L, Ag NPs at 5, 7.5 or 10 mL/L and K_2_Si_2_O_5_ at 0.5, 1 and 2 mg/L, with the respect of control (Table 8). The highest increments in the previous measurements were accompanying to the foliar spraying of Se at 0.1 mg/L, Ag NPs at 10 mL/L and K_2_Si_2_O_5_ at 1 or 2 mg/L over the other applied treatments and control. Additionally, the current results exhibited that K_2_Si_2_O_5_ at 2 mg/L was the superior treatment compared with the rest applied treatments. These results are in agreement with the past results of^107^, where the external spraying of selenium increased the content from vitamin C in tea. As Se increases the fruit content from TSS which contains acids, salts, vitamins, amino acids, sugars so, it can improve the taste of pear fruits^100^. Treating grape with Se boosted markedly the percentages of soluble sugar, soluble protein, soluble solid, as well as vitamin C, while it minimized organic acid percentage^6^. Selenium application on apple cv. Starking Delicious at 0.5, 1, and 1.5 mg/L twice during the enlargement fruit period improved remarkably soluble solid content^91^.

**Table 8:** Influence of the foliar spraying of Se, Ag NPS and K_2_Si_2_O_5_ on fruit content from total, reduced and non- reduced sugars, and anthocyanin of pomegranate cv. Succary during 2019 and 2020 seasons.

Treatments	Total sugars %		Reduced sugars %		Non-reduced sugars %		Anthocyanin (mg/100 g)	
	2019	2020	2019	2020	2019	2020	2019	2020
Control	8.99h	9.24h	6.59g	6.82h	2.40f	2.42e	0.32g	0.35g
0.025 mg/L Se	9.78g	10.22g	7.30f	7.49g	2.48f	2.73d	0.35fg	0.38g
0.05 mg/L Se	11.00e	11.11e	8.21de	8.21e	2.79d	2.90d	0.45d	0.44f
0.1 mg/L Se	13.04c	13.97b	9.74c	10.11b	3.3b	3.86a	0.49c	0.53c
5 mL/L Ag NPs	10.02g	10.71f	7.47f	7.88f	2.55ef	2.83d	0.36f	0.39g
7.5 mL/L Ag NPs	11.28e	11.76d	8.47d	8.56d	2.81d	3.20bc	0.45d	0.48de
10 mL/L Ag NPs	13.68b	14.05b	10.12b	10.33b	3.56a	3.73a	0.55b	0.59b
0.5 mg/L K_2_Si_2_O_5_	10.62f	10.80f	7.89e	7.85f	2.72de	2.95cd	0.40e	0.45ef
1 mg/L K_2_Si_2_O_5_	12.54d	13.21c	9.46c	9.79c	3.08c	3.42b	0.46cd	0.51cd
2 mg/L K_2_Si_2_O_5_	14.23a	14.55a	10.61a	10.64a	3.62a	3.91a	0.66a	0.69a
LSD_0.05_	0.36	0.21	0.33	0.27	0.21	0.27	0.03	0.03

Means not sharing the same letter(s) within each column are significantly different at 0.05 level of significance.

Low concentrations of Ag NP caused increasing of soluble sugar^16,77^. The foliar applications of Ag NPs on cucumber increased TSS of fruit^79^. Sprinkle of Ag NPs on sunflower at 50 mL/L increased the leaf content from carbohydrate as compared to control^16^. The external application of Ag NPs at 20 ppm on onion increased total soluble solids^15^. Ag NPs improved greatly the percentages of TSS, TSS/acid ratio, total, reduced and non-reduced sugars as well as fruit content from anthocyanin, while they reduced the fruit acidity percentage^82^.

The exogenous sprinkle of grapevines cv. Flame seedless with 250 ppm potassium silicate increased statistically the percentages of TSS, as well as total and reducing sugars compared with control^41^. Spraying of potassium silicate on ‘Grandnaine’ banana at 0.05 to 0.1% improved the percentages of TSS and total sugars, while it reduced the fruit acidity percentage compared to control^38^. Silicon application improved fruit content from total soluble solids, TSS /acid ratio and vitamin C in “Anna” apple^104^, and in orange cv. Olinda Valencia^88^. The external application of K_2_Si_2_O_5_at 0.05, 0.1 and 0.2% on ‘Keitte’ mango trees at the start of growth, just after fruit setting and 1 month later increased the percentages of TSS, total, reducing, non-reducing, vitamin C content, while it reduced the fruit acidity^87^. Treating of loquat trees with K_2_Si_2_O_5_at 1 or 2% improved the percentages of TSS, TSS/acid ratio, total sugars, vitamin C, while they lessened total fruit acidity proportion^43^. The external application of K_2_Si_2_O_5_ at 5000 ppm boosted the fruit chemical characteristics in terms of ascorbic acid, anthocyanin and total soluble solids percentages, while reduced the fruit acidity in pomegranate cvs. Wonderful^97^ and Manfalouty^98^ compared with untreated trees. External application of “Washington Navel” orange trees with and K_2_Si_2_O_5_at 0.2 and 0.3% boosted markedly the fruit content from the percentages of SSC, reducing, total, non-reduced, Vitamin C content, SSC/acidity ratio, while reduced the total fruit acidity %^89^.

The results listed in Table 9 showed that the foliar application of Se, Ag NPs and K_2_Si_2_O_5_ improved significantly the leaf mineral composition from N, P, and K macronutrients compared with the control during the two seasons. Additionally, the foliar application of 0.1 mg/L Se, 10 mL/L Ag NPs and 1 and 2 mg/L K_2_Si_2_O_5_ exhibited the high percentages from N, P and K compared with the other applied treatments in both experimental seasons. Moreover, the superior treatment was the application of K_2_Si_2_O_5_ at 2 mg/L. Low dose of Se can help in the balance of the necessary nutrient elements in the plants^5,70^. Spraying *Citrus Sinensis* with Se at 20, 40, 80 and 160 ppm improved obviously potassium, nitrogen and phosphorus concentration in the leaves with the respect of control and 40 ppm was the supreme one^69^.The application of nano fertilizers can help the translocation process of nutrients to the desired parts of plant^108^. Nano fertilizers greatly enhanced the uptake of NPK nutrients, which play an important role in increasing the growth, chlorophyll content and yield of wheat^80^.

**Table 9:** Influence of the foliar spraying of Se, Ag NPs and K_2_Si_2_O_5_ on the leaf composition from nitrogen, phosphorus, and potassium percentages of “Succary” pomegranate during 2019 and 2020 seasons.

Treatments	N %		P %		K%	
	2019	2020	2019	2020	2019	2020
Control	1.44e	1.47g	0.45f	0.55e	1.21e	1.36e
0.025 mg/L Se	1.75d	1.73f	0.49ef	0.56e	1.24e	1.39e
0.05 mg/L Se	1.80bcd	1.84de	0.59d	0.73cd	1.29cde	1.61cd
0.1 mg/L Se	1.85ab	1.92c	0.89c	0.84bc	1.41b	1.66c
5 mL/L Ag NPs	1.76cd	1.78ef	0.56de	0.60de	1.27de	1.50d
7.5 mL/L Ag NPs	1.82abc	1.84d	0.61d	0.83bc	1.38bcd	1.64c
10 mL/L Ag NPs	1.87a	1.98b	1.07b	0.91b	1.44b	1.79b
0.5 mg/L K_2_Si_2_O_5_	1.79bcd	1.79ef	0.58de	0.63de	1.28de	1.59cd
1 mg/L K_2_Si_2_O_5_	1.84ab	1.89cd	0.81c	0.84bc	1.40bc	1.65c
2 mg/L K_2_Si_2_O_5_	1.88a	2.06a	1.23a	1.19a	1.61a	1.90a
LSD_0.05_	0.06	0.05	0.09	0.14	0.10	0.10

Means not sharing the same letter(s) within each column are significantly different at 0.05 level of significance.

Spraying ‘grapevines with K_2_Si_2_O_5_ at 250 ppm four times boosted leaf mineral composition from N, P and K in the two seasons rather than control^41^.External application of K_2_Si_2_O_5_ at 0.05, 0.1 and 0.2% on ‘Keitte’ mango trees at the start of growth, after fruit setting and 1 month later raised the percent’s leaf P, K and Mg^87^. External application of orange with K_2_Si_2_O_5_ raised leaf N, P, and K, contents and K_2_Si_2_O_5_ at 4.0% the premier for leaf N and K content^88^.

### Antifungal activity

**Figure 1:**
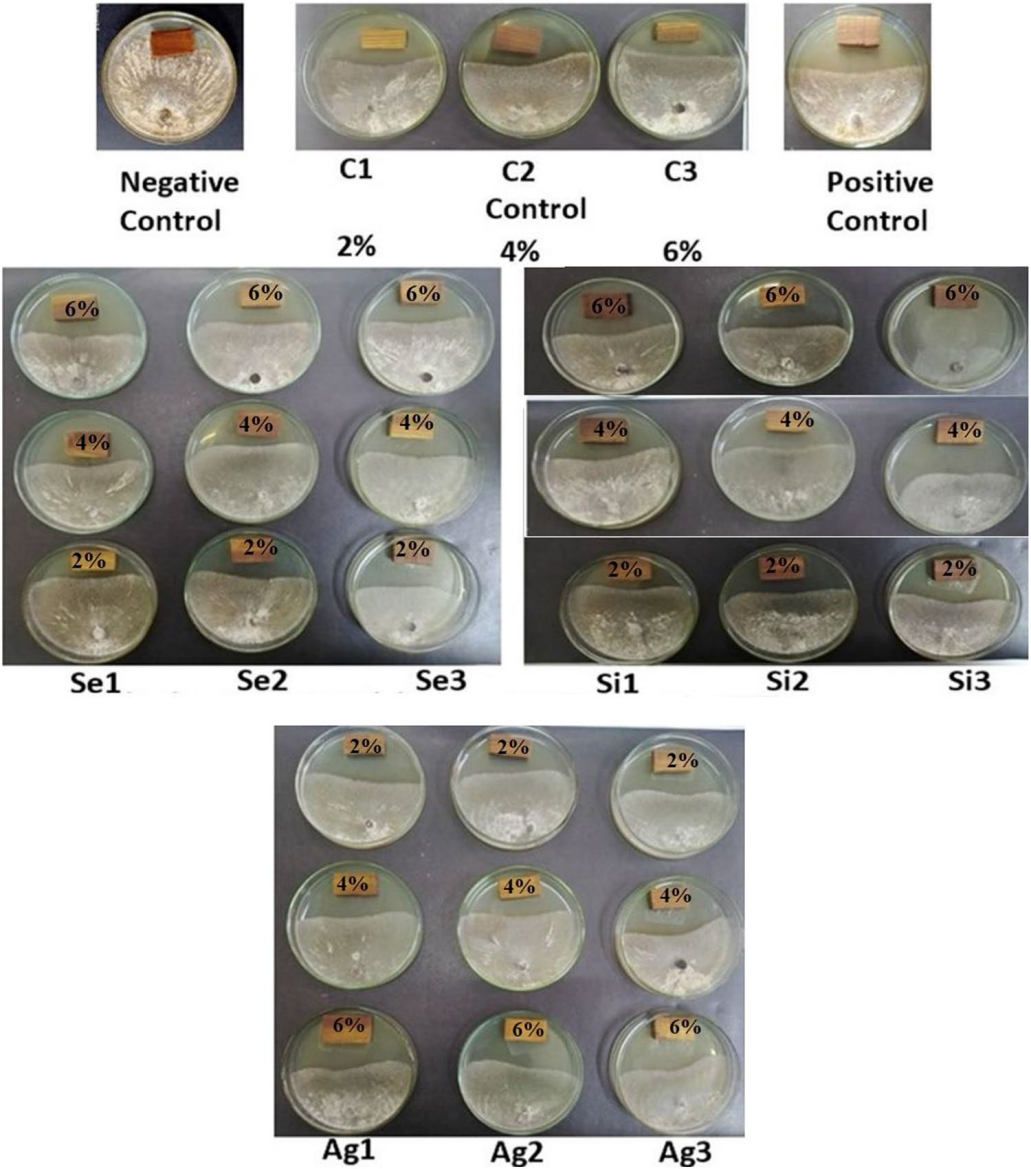
The visual observation of antifungal activity of wood-treated with acetone extracts from pomegranate peels collected from trees sprayed with different doses of Se1: 0.025 mg/L; Se2: 0.05 mg/L; Se3: 0.1 mg/L; Ag1: 5 mL/L; Ag2: 7.5 mL/L; Ag3: 10 mL/L; Si1: 0.5 mg/L; Si2: 1 mg/L; Si3: 2 mg/L, and untreated trees (C1, C2, C3).

Figure 1 shows the visual observation of the antifungal activity of wood-treated with peels’ acetone extracts from fruits of pomegranate as sprayed with numerous nanoparticles. Antifungal action of wood-treated with numerous acetone extracts from peels of pomegranate fruits as treated with variant nanomaterials in terms of inhibition percentages is shown in Table 1. The results cleared that the acetone extracts at the concentration of 6% from peels sprayed with 1 mg/L K_2_Si_2_O_5_, 0.025 mg/L Se, 2 mg/L K_2_Si_2_O_5_, 0.05 mg/L Se, 0.1 mg/L Se, 0.5 mg/L K_2_Si_2_O_5_ and 5 mL/L Ag NP, with inhibition 63.70, 51.48, 50.3, 48.14, 48.14, and 47.03, and 45.92%, respectively, opposed to *R. solani* compared to the positive control used (hesta 2.5 g/L) which noticed prevention values of 32.96%. Also, it can be shown from Table 10 that at the concentration of 4%, the acetone extracts from peels of fruits collected from trees treated with 0.025 mg/L Se, 1 mg/L K_2_Si_2_O_5_, and 0.05 mg/L Se showed inhibition proportions of 45.92, 44.81, and 43.70%, respectively, opposed to the growth of *R. solani*.

**Table 10:** Antifungal activity of acetone extracts from peels of pomegranate fruits collected from trees sprayed with different nanomaterials against the growth of *R. solani.*

Acetone extract from peel of fruits treated with	Extract concentration (%)	*R. solani* fungal inhibition percentage
Untreated	2%	35.18 ± 0.64
	4%	36.66
	6%	39.25 ± 0.64
5 mL/L Ag	2%	32.96 ± 0.64
	4%	39.25 ± 0.64
	6%	45.92 ± 0.64
7.5 mL/L Ag	2%	32.96 ± 0.64
	4%	32.59 ± 0.64
	6%	40.37 ± 0.64
10 mL/L Ag	2%	37.03 ± 0.64
	4%	38.51 ± 0.64
	6%	40
0.025 mg/L Se	2%	42.59 ± 1.69
	4%	45.92 ± 0.64
	6%	51.48 ± 0.64
0.05 mg/L Se	2%	39.25 ± 0.64
	4%	43.70 ± 1.28
	6%	48.14 ± 0.64
0.1 mg/L Se	2%	38.14 ± 0.64
	4%	41.11
	6%	48.14 ± 0.64
0.5 mg/L K_2_Si_2_O_5_	2%	33.33
	4%	40.74 ± 0.64
	6%	47.03 ± 0.64
1 mg/L K_2_Si_2_O_5_	2%	40.37 ± 0.64
	4%	44.81 ± 0.64
	6%	63.70 ± 0.64
2 mg/L K_2_Si_2_O_5_	2%	37.03 ± 0.64
	4%	39.25 ± 0.64
	6%	50.37 ± 0.64
Positive control*	2.5 g/L	32.96 ± 0.64
Negative Control	0	0.00
*P*-value		< 0.0001

### Phenolic compounds analysis of peel extracts

Data in Table 11 demonstrate that the greatest abundant concentration of catechol was found in acetone extract from peels of fruits treated with 0.05 mg/L Se (12.30 µg/mL) and 5 mL/L Ag NP (8.22 µg/mL) but not noticed in other treatments. Syringic acid was found with high quantity in acetone extract peels of fruits treated with 7.5 mL/L Ag NP (13.30 µg/mL), 0.025 mg/L Se (11.26 µg/mL) and 0.1 mg/L Se (8.66 µg/mL). The highest amount of *p*-coumaric acid was observed in acetone extract from peels of fruits treated with 0.1 mg/L Se (9.56 µg/mL), 7.5 mL/L Ag NP (9.55 µg/mL), 0.05 mg/L Se (9.22 µg/mL) and in untreated (6.77 µg/mL). Benzoic acid was identified only in acetone extracts of peels from fruits treated with 5 mL/L Ag NP (9.66 µg/mL), 10 mL/L Ag NP (5.47 µg/mL), 0.5 mg/L Si (5.98 µg/mL) and in untreated fruits (5.98 µg/mL).

**Table 11:** HPLC analysis of phenolic compounds in acetone extract of pomegranate fruit peels treated with different treatments.

Compound	Phenolic Compounds (µg/mL) in acetone extract of pomegranate fruit peels treated with									
	untreated	5 mL/L Ag NPs	7.5 mL/L Ag NPs	10 mL/L Ag NPs	0.025 mg/L Se	0.05 mg/L Se	0.1 mg/L Se	0.5 mg/L K_2_Si_2_O_5_	1 mg/L K_2_Si_2_O_5_	2 mg/L K_2_Si_2_O_5_
Catechol	ND*	8.22	ND	ND	ND	12.30	ND	ND	ND	ND
Syringic acid	6.13	5.14	13.30	4.66	11.26	ND	8.66	4.66	4.11	2.69
*p*-Coumaric acid	6.77	ND	9.55	ND	ND	9.22	9.56	ND	ND	ND
Benzoic acid	5.98	9.66	ND	5.47	ND	ND	ND	5.98	ND	ND
Caffeic acid	ND	4.15	3.25	7.88	12.06	ND	ND	ND	ND	10.77
Pyrogallol	10.23	ND	ND	2.19	5.16	ND	12.39	2.74	12.69	ND
Gallic acid	9.44	5.04	ND	12.44	ND	ND	ND	ND	2.06	3.49
Ferulic acid	3.12	ND	6.12	ND	13.09	5.12	4.16	ND	ND	11.97
Salicylic acid	ND	6.18	6.23	ND	ND	11.45	3.88	12.44	3.26	ND
Cinnamic acid	19.36	14.33	5.14	3.69	4.36	ND	ND	3.19	ND	0.87
Ellagic acid	ND	4.19	ND	ND	ND	18.33	7.69	ND	ND	ND

*ND not detected.

**Figure 2:**
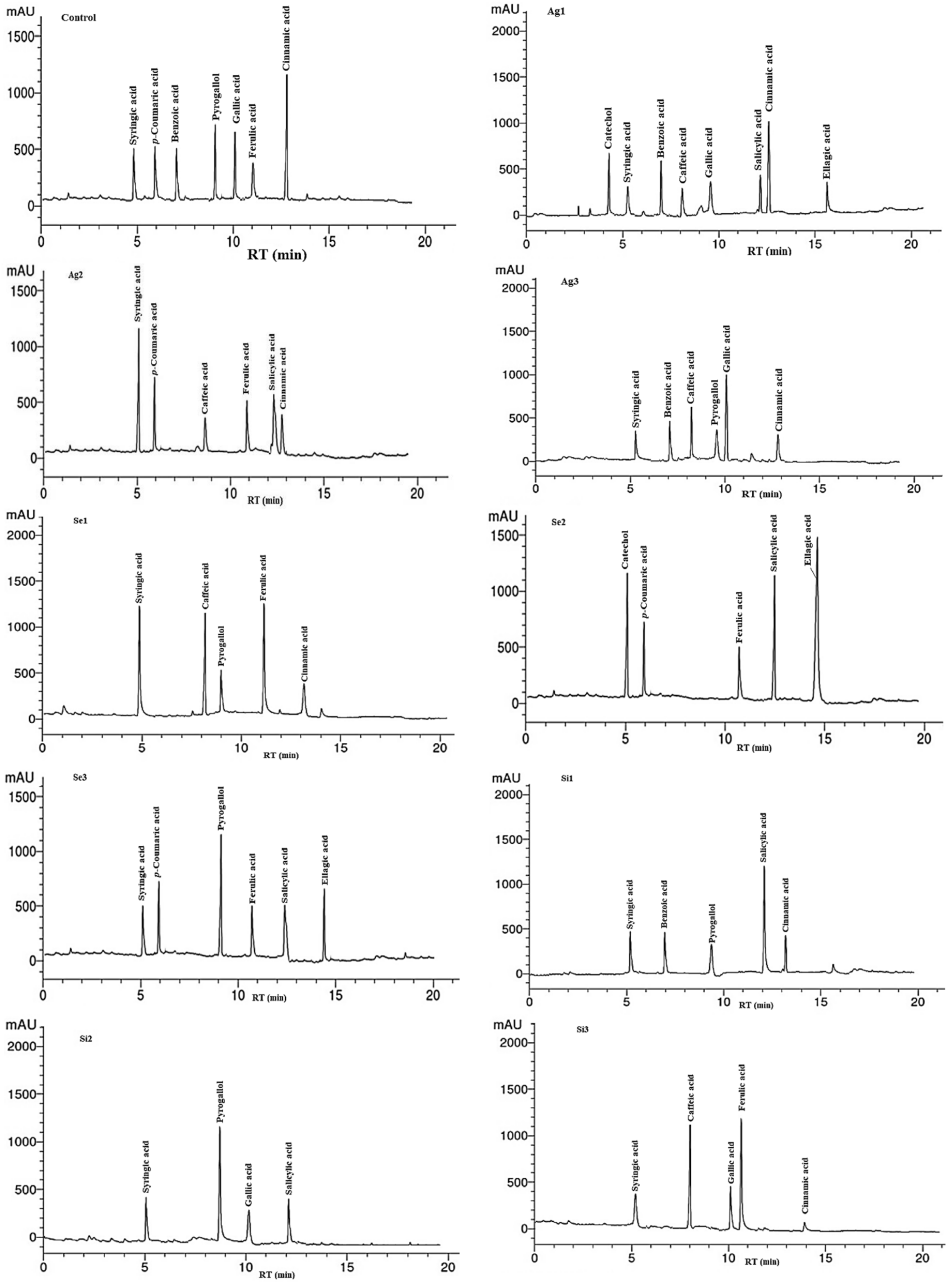
HPLC chromatograms of phenolic compounds from acetone extracts of pomegranate peels fruits treated with Ag1: 5 mL/L Ag NPs; Ag2: 7.5 mL/L Ag NPs; Ag3: 10 mL/L Ag NPs; Se1: 0.025 mg/L Se; Se2: 0.05 mg/L Se; Se3: 0.1 mg/L Se; Si1: 0.5 mg/L K_2_Si_2_O_5_; Si2: 1 mg/L K_2_Si_2_O_5_; Si3: 2 mg/L K_2_Si_2_O_5_.

The highest concentration of caffeic acid was seen in acetone extract from peels of fruits treated with 0.025 mg/L Se (12.06 µg/mL), 2 mg/L K_2_Si_2_O_5_ (10.77 µg/mL) and 10 mL/L Ag NP (7.88 µg/mL). Pyrogallol compound showed the highest abundant concentrations in acetone extract from peels of fruits collected from trees sprayed with 0.1 mg/L Se (12.39 µg/mL) and 1 mg/L K_2_Si_2_O_5_ (12.69 µg/mL) and in untreated (10.23 µg/mL). Gallic acid with high amount was observed in acetone extract from peels of fruits from trees treated with 10 mL/L Ag NP (12.44 µg/mL) and in untreated (9.44 µg/mL). The highest amount of ferulic acid was identified in acetone extract from peels of fruits treated with 0.025 mg/L Se (13.09 µg/mL), 0.1 mg/L K_2_Si_2_O_5_ (11.97 µg/mL) and 7.5 mg/L Ag NP (6.12 µg/mL). Salicylic acid was found at high quantity in acetone extracts from peels of fruits treated with 0.5 mg/L K_2_Si_2_O_5_ (12.44 µg/mL) and 0.05 mg/L Se (11.45 µg/mL). Cinnamic acid showed the highest amount in acetone extracts from fruit peels of trees treated with 5 mL/L Ag NP (14.33 µg/mL) and untreated trees (19.36 µg/mL). The highest concentration of ellagic acid was observed in acetone extracts from peels of treated trees with 0.05 mg/L Se (18.33 µg/mL) and 0.1 mg/L Se (7.69 µg/mL). The full HPLC chromatograms of the isolated and identified phenolic compounds are shown in Fig. 2.

Therefore, the bioactivities of acetone treated-wood are linked to the existence of bioactive phenolic compounds. Peel extract has been reported to contain more phenolics than seed or pulp extracts^46,109^. The predominant compounds were gallic acid, ellagic acid, quercetin, caffeic acid, *p*-coumaric acid, and vanillic acid were found in peel extract^110,111^. Gallic and ellagic acids were presented in the methanolic extract of pomegranate peel^112^. Peel extracts demonstrated power antifungal activity against *Aspergillus niger* and *Trichoderma reesei*^113^. Peel extract showed markedly antifungal activity against *A. parasiticus* and *A. parasiticus*^114^. Peel extract noticed high activity against *A. parasiticus* and no activity against *A. flavus*^115^. The development levels of *Alternaria alternata*, *Stemphylium botryosum*, and *Fusarium* spp. growth rates were significantly inhibited by the peel extracts^116^ that were negatively linked to the rates of punicalagins, the major ellagitannins in pomegranate peels. *F. oxysporum* mycelia development was prevented by 62% (propanol extract) to 78% (water extract) of peel extracts^117^. The current study is in harmony with our previous works related to wood-biofungicides. Acetone extracts of *Acer saccharum var. saccharum* inner and outer bark in combination with citric acid when applied to wood showed strong bioactivities against *Trichoderma viride*, *Fusarium subglutinans*, and *A. niger*^56^, where, phenolic compounds p-hydroxy benzoic acid, gallic acid, salicylic acid, vanillin and o-coumaric acid, and ferulic acid were identified by HPLC. Wood samples of *M. azedarach* wood treated with flower aqueous extract of *Acacia saligna* showed good antifungal activity against *Penicillium chrysogenum* and moderate activity against *Fusarium culmorum* and *Rhizoctonia solani*^59^, and the HPLC showed the presence of benzoic acid, caffeine, and o-coumaric acid as most abundant compounds. *F. culmorum* and *R. solani* mycelial growths were inhibited significantly as wood treated with 3% methanolic extract of *Musa paradisiaca* peel that showed phenolic compounds of ellagic acid and gallic acid^118^.

## Conclusion

Spraying Se, Ag NPs and K_2_Si_2_O_5_ raised the shoot length_,_ diameter, leaf chlorophyll, fruit set proportion, and fruit yield, fruit weight, length and diameter, total soluble solids, total, reduced and non-reduced sugars, while they minimized the juice acidity percent with the respect to control during our study. Fruit cracking and fruit sunburn were lessened markedly by the application of Se, Ag NPs and K_2_Si_2_O_5_ with respect to control. The application of 0.1 Se mg/L, 10 ml/L Ag NPs and 2 mg/L K_2_Si_2_O_5_ was more effective than 0.025 or 0.05 mg/L Se, 5 or 7.5 mL/L Ag NPs and 0.5 or 1 mg/L K_2_Si_2_O_5_ in improving the developing performance, yield and yielding components through studying times. The supreme treatments, which achieved the best results, was the application of 2 mg/L K_2_Si_2_O_5_ over the other applied treatments during our study seasons. Additionally, the extracts from fruit peels identified several bioactive phenolic compounds and the extracts observed good wood-biofungicide.

## Data Availability

All data generated or analyzed during this study are included in this published article.
